# The relationship between the parenteral dose of fish oil supplementation and the variation of liver function tests in hospitalized adult patients

**DOI:** 10.1186/s12937-015-0048-6

**Published:** 2015-07-02

**Authors:** Maria B. Badia-Tahull, Elisabet Leiva-Badosa, Ramon Jodar-Masanes, Josep Maria Ramon-Torrell, Josep Llop-Talaveron

**Affiliations:** 1 Pharmacy Department, Hospital Universitari Bellvitge. IDIBELL. C/Feixa Llarga s/n. 08907 L’Hospitalet de Llobregat, Barcelona, Spain; 2 Preventive Medicine Department, Hospital Universitari Bellvitge. IDIBELL. C/Feixa Llarga s/n. 08907 L’Hospitalet de Llobregat, Barcelona, Spain

**Keywords:** Liver function tests, Fish oil emulsion, Fatty acids, Omega-3 fatty acids, Parenteral nutrition

## Abstract

**Background:**

Hepatic dysfunction is a complication associated with parenteral nutrition (PN). Our primary objective was to study the relationship between doses of intravenous fish oil (FO) emulsion in PN and the variation in the main liver function tests (LFTs) in hospitalized PN-treated adults. As a secondary objective, we studied the safety of FO administration.

**Methods:**

We conducted a retrospective study in adult patients receiving FO supplementation in PN. Demographic, nutritional and safety variables were collected. Variation of LFTs was defined as the difference between values just before the first administration of FO and values at the end of PN. A multiple linear regression was performed to study the association between PN-lipids (FO or vegetable) and the variation of each LFT; the following variables were used to adjust the effect of lipids: sepsis, length of stay in the intensive care unit and lipids dose. Student t-test was used to study safety variables. Data were analyzed using SPSS 19.0.

**Results:**

Patients (53, median age 68 years (24–90); 62 % men) with the principal diagnosis of digestive neoplasm (42 %) received PN for a median of 19 (7–75) days. In the multivariate analysis, the amount of FO was related to a decrease in gamma-glutamyl transferase (GGT) (B = −2.23;CI95 % = −4.41/-0.05), a decrease in alkaline phosphatase (AP) (B = −1.23;CI95 % = −2.07/-0.37), and a decrease in alanine aminotransferase (ALT) (B = −0.82; CI95 % = −1.19/-0.44). No differences were found in safety variables.

**Conclusions:**

GGT, AP and ALT improved with FO PN-supplementation. Moreover, the improvement was greater when the doses of FO were higher. FO administration in PN is safe.

## Background

Fat is increasingly recognized as a central feature of many biological processes [1]. Lipid emulsions are an essential part of parenteral nutrition (PN), both as an energy supply and as a source of essential fatty acids (FAs) [2]. Furthermore, they are involved in the structure and function of cell membranes and receptors, modifying gene expression and modulating the inflammatory and immune responses [2]. Moreover, FAs are precursors of prostaglandins and other eicosanoids and have therefore important metabolic functions [2].

The development of significant hepatic dysfunction is a well-recognized complication associated with PN administration [2, 3]. A spectrum of hepatobiliary disorders, ranging from simple steatosis to cholestasis, cholelithiasis, hepatic fibrosis and subsequent progression to cirrhosis, portal hypertension and end-stage liver disease, can occur [4]. This disorder is characterized by elevated serum levels of total bilirubin (BIL), alkaline phosphatase (AP), gamma-glutamyl transferase (GGT) and transaminases (alanine aminotransferase [ALT] and/or aspartate aminotransferase [AST]) [2, 3, 5]. Cholestasis and hepatocellular damage are well-known complications caused by long-term PN, especially in the neonatal and pediatric population but also in adult patients with short bowel syndrome (SBS). In adult patients, this hepatic dysfunction may be present as cholestasis, hepatosteatosis or cholelithiasis [5]. Parenteral nutrition-associated liver disease (PNALD) is well established in patients with long-term home PN treatment. But it is also true that those patients who only receive PN during hospitalization have the potential to develop alterations in their LFTs [6, 7]. Although liver failure develops slowly over months or years, steatosis and steatohepatitis may develop within weeks in acutely ill patients [4]. This liver alteration is normally transient, and may not progress to a worse condition. However, it could add to those liver alterations caused by drugs and specific clinical situations and might also pose a limitation to a correct intravenous nutrient supply.

The mechanisms leading to PNALD remain largely unknown but are likely to be multifactorial [2, 5, 8]. Established risk factors for its development are duration of PN and number of septic episodes [9], but several aspects of the parenteral formula have also been thought to be responsible for these liver test abnormalities [10, 11]. Macronutrient excess, i.e., feeding beyond the liver’s ability to use carbohydrate and fat, can cause accumulation of by-products in the liver and result in hepatic injury. Another cause is the source of fat used. Lipids coming from soybean oil (SO) have high levels of ω-6 and are associated with liver toxicity, mainly with long-term utilization. After them, median chain triglycerides (MCT) were combined with SO in order to provide energy quickly and avoid some of the secondary effects detected with SO. Olive oil-based lipids were introduced later with potentially fewer lipoperoxidation problems; and recently, lipids derived from fish have been reported to have anti-inflammatory activity. Combinations of all these lipids have appeared on the market and their use has been extended. The long-term use of these emulsions has permitted their correlation with different types of PNALD and with recovery from PNALD in premature and home parenteral nutrition patients. Initially, in our hospital, only SO was used but, as soon as different products for inflammatory conditions were marketed, we also introduced them in our clinical protocol. These decisions were based on clinical experience, coming mainly from the clinical guidelines of the European Society of Parenteral and Enteral Nutrition (ESPEN). In addition, vegetable oil-based lipid emulsions contain significant quantities of phytosterols that accumulate in patients’ serum and result in cholelithiasis [10]. Long-term use of soybean oil (SO)-based emulsions leads to a progressive increase of phytosterol content in cell membranes and plasma lipoproteins which has been associated with the onset of PNALD in children receiving long-term PN [9]. SO also depletes vitamin E from plasma lipoproteins and may contribute to hepatic injury [12]. Inflammation is another crucial factor and ω-6 long chain polyunsaturated fatty acids (PUFAs) have been shown to worsen inflammatory states and to have immunosuppressive effects [9]. The peroxidative nature of ω-6 could increase inflammatory activity in an infectious (sepsis) or non-infectious episode [4].

Some evidence has suggested that the administration of FO-based lipid emulsions may be useful in reversing PNALD [13]. These emulsions are primarily composed of ω-3 PUFAs. In Europe, FO can be used both as part of a standard lipid emulsion or as a supplement, alone or in combination with other lipid emulsions. FO is mostly used to attenuate the patient’s inflammatory state and not to modify liver function. The purpose of this study was to test, in routine clinical practice, whether there is an influence of FO on LFTs in adult patients treated with PN during hospitalization.

Our main objective was therefore to study the relationship between the doses of intravenous FO emulsion supplementation and the variation in the main LFTs in adult patients receiving PN during hospitalization. As a secondary objective, we studied the safety of the doses of FO given.

## Methods

This was a retrospective study conducted over a 16-month period in a 600-bed, third-level teaching hospital. Patients included in the study met the following criteria: older than 17 years of age, receiving FO supplementation in PN and not receiving enteral nutrition or oral food. Those patients with liver disease previous to PN initiation were excluded.

Data were collected in several categories: demographic variables (gender, age, height and weight); clinical variables (diagnosis, indication for PN administration, sepsis episodes, length of stay [LOS] in intensive care unit [ICU] and death); and nutritional variables (length of treatment with PN and amount of parenteral macronutrients administered). Nutritional data were collected from the database program of PN in the Pharmacy Department. Analytical and clinical data were collected from the hospital’s computerized database.

Lipids were given as: Clinoleic® (Baxter, Lessines, Belgium) based on a mixture of purified olive oil (80 %) and SO (20 %); SMOFlipid® (Fresenius Kabi, Bad Homburg, Germany) based on SO (30 %), MCT (30 %), olive oil (25 %) and FO (15 %); and Omegaven® (Fresenius Kabi, Bad Homburg, Germany), an emulsion exclusively of FO. In our study the amount of lipids administered by source, either vegetable (SO, MCT, olive oil) or not (FO), was studied separately.

In our routine practice, according to our protocol, lipids in PN are administered as a maximum dose of 1 g/kg/day, although this dose is reduced in situations of hypertriglyceridemia. Primarily, when the patient presents an inflammatory state, SMOFlipid® is used. However, in those patients with a high inflammatory state a combination of Omegaven® and Clinoleic® is used in order to achieve higher proportions of FO (with a maximum of 50 % of the dose). To classify the inflammatory state, we used C-reactive protein (CRP) [mg/L] and prealbumin [mg/L] as biochemical and immunology parameters, since they are acute phase proteins. Nevertheless, in the extreme situation, even when the prealbumin is not so low, we maintained high proportions of omega-3 if CRP values were high. A third parameter that we have taken into account is plasma triglycerides [mmol/L] because of the better clearance of omega-3 lipids [4]. In addition to these parameters we have also taken into account the clinical situation of the patient. In cases of hypertriglyceridemia where the total dose is reduced, lipids are given only as Omegaven®. Once FO emulsion was introduced in the nutritional formula, it was continued until the end of PN treatment. So, the same patient could receive different types of lipids according to their clinical situation All patients studied received a fixed standard daily supplementation of trace elements and vitamins.

### Assessment

Venous blood samples were taken at the beginning of treatment with PN containing FO and twice a week thereafter. Liver function assessment was carried out with measurements of GGT, AP, ALT and BIL. On the other hand, safety variables were only determined at the beginning of the study and at the end of PN treatment; these variables were serum triglyceride (TG) levels and markers of coagulopathy: platelets count (PLC) and the international normalized ratio (INR).

### Statistical analysis

For the statistical analysis, the principal variable chosen was the variation of LFTs. It was defined as the difference between LFT values just before the first administration of FO in the PN and the final values at the end of PN administration (results of these differences could be positive or negative according to an elevation or a diminution in the parameter, respectively).

Using this principal variable, two different statistical models were constructed:

1. Univariate model
  The association between the variation in the liver parameters and the total dose of FO administered during the study (g/kg) was evaluated by one-way analysis of variance (ANOVA). Five categories were defined according to the variation found in each liver parameter: the values of the variations obtained were classified as high increase (including patients above with an increase superior to the 80^th^ percentile), moderate increase (from the 60^th^ to the 80^th^ percentile), no modification (between the 40^th^ and 60^th^ percentile), moderate decrease (20^th^ and 40^th^ percentile) and high decrease (with a variation of less than the 20^th^ percentile).
2. Multivariate model
  A multiple linear regression model was constructed to study the relationship between each LFT variation as a continuous variable and the amount of FO given adjusted in accordance with four important variables of liver co-morbidity:
  Presence of sepsis when each liver parameter presented the highest value. According to the American College of Chest Physician/Society of Critical Care Medicine (ACCP/SCCM) consensus conference, sepsis was established when the patient presented at least two of the following criteria: (a) body temperature ≥38 °C or ≤36 °C, (b) tachycardia with heart rate ≥90 beats/min, (c) tachypnea with paCO_2_ ≤ 32 mmHg or mechanical ventilation; (d) leukocytes >12x10^9^cells/L [14].
  LOS in ICU considered as the number of days that the patient stayed in ICU.
  Dose of lipids. This variable contained two different elements: FO and vegetable-based lipids. In order to avoid the co-linearity effect between them, the amount of FO was introduced in the statistical model adjusted by residuals.

Additionally, in order to check the safety of FO administration, differences between the beginning and the end values for safety variables (TG, PLC and INR) were studied with t-test for paired samples.

All data were analyzed using SPSS 19.0 (SPSS INC, Chicago IL, USA). Statistical significance was reported with a 95 % confidence interval at the conventional p < 0.05 level (two-tailed).

Written informed consent was considered not necessary for the study, as it was a non-interventional study. Parenteral nutrition and nutritional and safety variables were prescribed and collected according to clinical practice. Patient data were anonymized for the purposes of this analysis. Confidential patient information was protected according to national normatives. This manuscript has been revised for publication by the Clinical Research Ethics Committee of our Hospital.

## Results

During the total study period (January 2010 to April 2011), 53 patients received PN-FO supplementation and were included in the study. Median age was 68 (24–90) years, median weight was 70 kg (40–158) and 62 % of participants were men. Patients received the standard drug treatment for their pathology.

The most frequent diagnosis was digestive neoplasm (42 %) (Table 1) and the main indication for PN administration was paralytic ileus or intestinal failure. Overall, 40 patients stayed in ICU with a median LOS of 9.5 days (2–99). Among the studied patients, 8 died during the follow-up period (15.1 %).

**Table 1:** Diagnosis

Diagnostic	n (%)
Digestive neoplasm	22 (42 %)
Pancreatitis	7 (13 %)
Other digestive diseases	6 (11 %)
Septic shock	5 (9 %)
Multiple trauma	4 (8 %)
Cardiovascular disease	3 (6 %)
Other neoplasm	3 (6 %)
Status epilepticus	2 (4 %)
Respiratory disease	1 (2 %)
*Total*	*53 (100 %)*

The median length of treatment with PN was 19 (7–75) days. In Table 2, the amount of nutrients is described. Altogether, types of lipids administered to the patients were: 24 % SO, 18.8 % MCT, 36.6 % olive oil, 20.6 % FO. The most frequent emulsion used to supply lipids was a combination of Clinoleic® with Omegaven®.

**Table 2:** Main nutritional and analytical variables collected during the study

Variables	Median (range)	
Body Mass Index (BMI)	25.4 (17.3–41.5)	
Parenteral Nutrition regimen		
Total non-protein calorie (kcal/kg/d)	16.7 (7.7–28.3)	
Proteins (g/kg/day)	1.04 (0.43–1.88)	
Total fat (g/kg/day)	0.61 (0.22–1)	
Fish oil emulsion (g/kg/day)	0.07 (0.01–0.9)	
Dextrose (g/kg/day)	2.73 (1.17–4.58)	
Laboratory	Initial PN	Final PN
Serum albumin (g/L)	28 [15–42]	30 [15–38]
Serum prealbumin (mg/L)	123 (17.6–209)	199 (41.1–290)
C-Reactive protein (mg/L)	186.4 (3.6–405.6)	133.7 (2.4–339.5)
Bilirubin (μmol/L)	8.25 (2–151)	7.0 (2–358)
Alanine aminotransferase (μkat/L)	0.32 (0.11–4.67)	0.55 (0.11–5.83)
Alkaline phosphatase (μkat/L)	1.4 (0.5–11)	3.0 (0.6–14.20)
Gamma-glutamyl transferase (μkat/L)	1.29 (0.17–8.51)	4.28 (0.16–46.72)
Triglycerides (mmol/L)	1.7 (1.1–3.6)	1.7 (1.0–3.4)
Platelet count (x10^9^cells/L)	315 (54–1285)	368 (30–834)
International normalized ratio (INR)	1.22 (0.94–2.56)	1.20 (0.78–1.94)

Among the 23 septic patients, 22 (95.6 %) presented their highest values of GGT, AP or ALT during the septic event; while 23 (100 %) had their highest value of BIL simultaneously with the sepsis.

### Statistical results

In the univariate model ANOVA, variation of LFTs was categorized and the number of patients classified in each group is shown in Table 3. ANOVA results showed that there were statistically significant differences regarding AP. Those patients with a higher decrease of AP had received higher doses of FO. The mean of the total dose received was 8.96 g/kg in those with a high decrease of AP.

**Table 3:** Analyses of variance between doses of fish oil and the variation of each liver parameter

Gamma-glutamyl transferase variation						
Variation category	High increase	Moderate increase	No modification	Moderate decrease	High decrease	*p*
n	10	8	9	19	7	0.156
FO dose mean^a^	1.69	0.64	1.08	3.61	4.47	
Rank	0.01–6.44	0.02–2.79	0.04–2.25	0.17–22.56	0.4–13.12	
**Alkaline phosphatase variation**						
Variation category	High increase	Moderate increase	No modification	Moderate decrease	High decrease	*p*
n	8	16	16	7	6	**0.000**
FO dose mean^a^	2.22	1.16	1.72	1.41	8.96	
Rank	0.01–6.44	0.02–2.79	0.05–12.6	0.3–3.6	3.36–22.56	
Alanine aminotransferase variation						
Variation category	High increase	Moderate increase	No modification	Moderate decrease	High decrease	*p*
n	7	10	23	7	6	0.094
FO dose mean^a^	0.66	0.57	2.73	3.77	5.32	
Rank	0.01–1.52	0.02–2.47	0.04–13.12	0.24–12.6	0.3–22.56	
Bilirubin variation						
Variation category	High increase	Moderate increase	No modification	Moderate decrease	High decrease	*p*
n	5	11	15	14	8	0.057
FO dose mean^a^	1.94	0.71	1.35	3.69	5.28	
Rank	0.3–6.44	0.02–1.63	0.02–3.6	0.22–13.12	0.03–22.56	

*FO* fish oil

*SD* Standard deviation

^a^Mean of the total dose of fish oil emulsion administered, expressed in g/kg

Figures marked in bold are statistically significant in the study

No significance was found between the total dose of FO and GGT; a trend towards significance was found in ALT and BIL (Table 3).

**Fig. 1:**
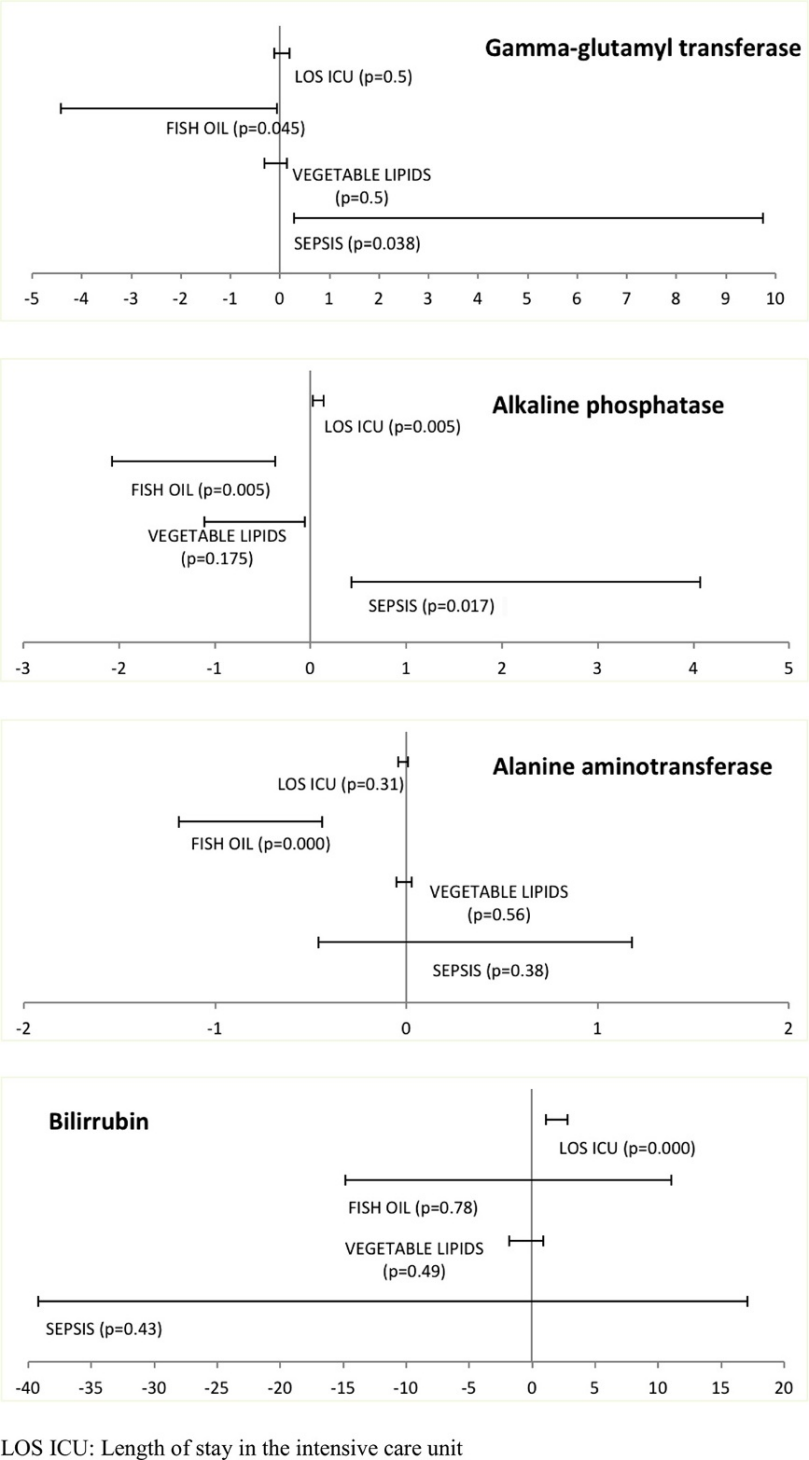
Representation of the B 95 % confidence interval of the four risk factors associated to each liver function test

In the multivariate model, variation of LFTs was considered as a continuous variable and the following statistically significant results were obtained (Table 4 and Fig. 1):

- The variable associated with an increase in GGT values was sepsis, B = 5.03 [CI 95 % = 0.29/9.76], however, the total amount of FO administered was associated with a decrease in GGT, B = −2.23 [CI 95 % = −4.41/-0.05].
- The increase of AP was associated with sepsis, B = 2.25 [CI 95 % = 0.43/4.07] and a greater number of days in ICU, B = 0.08 [CI 95 % = 0.03/0.14]. On the other hand, the total amount of FO administered was associated with a decrease in AP, B = −1.23 [CI 95 % = −2.07/-0.37].
- ALT values only showed association with one variable: ALT decreased when the dose of FO administered increased B = −0.82 [CI 95 % = −1.19/-0.44].
- Finally, increases in BIL were only associated with a longer stay in ICU B = 1.97 [CI 95 % = 1.11/2.84].

**Table 4:** Multiple lineal regressions between liver parameters variation and four factors that can affect them

Liver parameter								
Variable	Gamma-glutamyl transferase		Alkaline phosphatase		Alanine aminotransferase		Bilirubin	
	B CI 95 %	*p*	B CI 95 %	*p*	B CI 95 %	p	B CI 95 %	*p*
Sepsis	**5.03 [0.29/9.76]**	**0.038**	**2.25 [0.43/4.07]**	**0.017**	0.36 [−0.46/1.18]	0.38	−11.04 [−39.2/17.1]	0.43
Total vegetable lipids dose	−0.077 [−0.31/0.152]	0.50	−0.03 [−1.11/0.06]	0.175	−0.11 [−0.05/0.03]	0.56	−0.47 [−1.82/0.88]	0.49
Total fish oil dose	**−2.23 [−4.41/-0.05]**	**0.045**	**−1.23 [−2.07/-0.37]**	**0.005**	**−0.82 [−1.19/ -0.44]**	**0.000**	−1.81 [−14.8/11.1]	0.78
Length of stay in the intensive care unit	0.05 [−0.10/0.20]	0.50	**0.08 [0.03/0.14]**	**0.005**	−0.01 [−0.04/0.01]	0.31	**1.97 [1.11/ 2.84]**	**0.000**
*Determination Coefficient*	R^2^: 0.15		R^2^: 0.33		R^2^: 0.31		R^2^: 0.34	

*CI* confidence interval

Figures marked in bold are statistically significant in the study

Determination coefficients (R^2^) were consistent.

In the t-tests for safety variables, no differences were found in the serum TG levels: initial TG median value 1.7 mmol/L (1.1-3.6) and final TG median 1.7 mmol/L (1.0-3.4) (p = 0.666). With regards to coagulation markers, no differences, but a trend to significance, was found in PLC and INR values between the beginning and the end of treatment. PLC initial median 315×10^9^ cells/L (54×10^9^-1285×10^9^) vs. final median 368×10^9^ cells/L (30×10^9^-834×10^9^) (p = 0.073); and INR initial median 1.22 (0.94-2.56) vs. final median 1.2 (0.78-1.194) (p = 0.059).

## Discussion

This study demonstrated that, when administering FO supplementation to adult patients treated with PN during hospitalization, higher doses of FO were associated with an improvement in some liver parameters (GGT, AP and ALT), regardless of other factors that are normally associated with the alteration of these LFTs in patients undergoing PN treatment.

To our best knowledge, little research has been conducted on the management of liver alteration in adults during hospitalized PN treatment with FO. In fact, very few studies have focused on adults with SBS and PNALD [13, 15], while other aspects of the potential benefits of Omegaven® in reducing infections and improving postoperative health outcomes in the critically ill population have been well studied [16].

LFT alterations associated with PN is a frequent finding and can be related to many factors [17], among them intravenous lipid administration [18] since in 1982, Allardyce [19] reported on the prevention of cholestasis with the use of lipid reduction strategies. The cause appears to be at least partially associated with the composition of the SO lipid emulsions that aggravates or induces hepatocellular and systemic inflammatory effects [4]. Lipid metabolism results in lipid peroxidation and free radical formation, causing functional damage to hepatocytes, leading to reduced bile production and cholestasis in animal models. The higher oxidation susceptibility of ω-6 PUFAs requires antioxidant enrichment to counteract formation of reactive oxygen species, although currently utilized levels may not be sufficient [9]. Following these concerns, several alternatives were investigated and, although the precise mechanism by which these effects are triggered has yet to be clearly determined, parenteral FO administration shows several advantages that could explain the improvement in liver function. FO lipid emulsion lacks hepatotoxic phytosterols [20, 21]; improves biliary flow and cholestasis; and it has been shown to be effective in lowering triglyceride levels by increasing their clearance [4, 22]. Owing to their high concentration of ω-3 PUFAs [20], FO has an anti-inflammatory potential via competitive inhibition of the ω-6 PUFAs-derived pro-inflammatory eicosanoids [23] linked to liver injury causing both apoptosis and necrosis in many types of liver disease (cholestasis, non-alcoholic fatty liver disease, alcoholic cirrhosis and hepatitis) [5]. That is why some authors propose, in line with our results, that increasing the hepatic content of ω-3 PUFAs could decrease inflammatory activity in acute hepatitis with alleviation of hepatic injury and inflammation [23]. Parenteral FO is enriched with high levels of the antioxidant α-tocopherol to counteract possible oxidative risk [9], while other lipids contain γ-tocopherol and have less anti-oxidative effects [24]. Finally, FO-containing emulsions improve splanchnic blood flow [2, 25, 26].

Clinically, the relationship between ω-3 FAs and liver function has been more intensively studied in children with SBS and PNALD. Since Gura et al. [27, 28] published their first papers on the subject, many publications have appeared showing different strategies to deal with this problem [29–32]. Currently, even if there is some concern that the improvement found in PNALD in children might be due to lipid dose reduction rather than the type of lipid administered [33] a systematic review by Seida et al. [34] concluded that the best available evidence at present supports the use of ω-3 FAs supplementation to improve biochemical outcomes of PNALD in young children dependent on PN.

There are some studies on FO administration in adult patients with SBS and PNALD. Xu et al. [13] in a 15-patient study, found that bilirubin normalized after 4 weeks of treatment when SO content of PN was partially replaced by FO up to 10 g daily, about 0.15-0.2 g/kg/d. Differences in ALT values were also statistically significant. Nevertheless, even when GGT values also decreased, statistically significant differences were not found. Venecourt et al. [35] showed that after changing one patient to a total FO emulsion, bilirubin decreased over the ensuing 8 weeks, with normalization of transaminases in 10 weeks. In the case of Burns et al. [36], FO administration decreased total bilirubin; AP initially increased but subsequently declined to a stable pretreatment value and transaminases slightly decreased initially and then remained stable. In a patient described by Jurewitsch et al. [37] the change to a blend of FO plus SO curtailed the increase in liver enzymes but did not reduce total bilirubin levels. It was subsequently decided to give only FO and total bilirubin levels fell rapidly. The main difference between those patients and ours is that all those patients had established liver disease when they started treatment with FO and they had developed liver disease after a long period of PN treatment: 110 days in the Jurewitsch study [37], 3 years in the Venecourt paper [35] and 5 years in Burns [36]. In the Xu study [13], patients developed PNALD after 2–19 months of treatment with PN. In our patients, we found a significant decrease in AP, GGT and ALT values, even when they had received PN for a short period of time (median of 19 days) and they did not achieve this status of an advanced form of liver disease. On the other hand, the main commercial emulsions that we used to provide lipids were the combination of Clinoleic® with Omegaven® and no studies yet show whether the immunoneutral effect of high amounts of olive oil could contribute to the improvement of some liver parameters.

Studies on LFT alterations and hospitalization-PN administration using FO in adults are scarce. Our results on ALT improvement with FO agree with those found in the bibliography. In patients undergoing liver transplantation, Zhu et al. [38] found that ALT improved significantly after 9 days of FO supplementation (0.2 g/kg/d) compared with traditional PN support (LCT:MCT). Total lipid intake was 1 g/kg/d. In a randomized, double-blind trial comparing SMOFlipid® with olive oil in patients following major abdominal surgery or large cranio-maxillo-facial resections for cancer, Piper et al. [2] found that significantly lower ALT values were observed at days 2 and 5 in the SMOFlipid® group. Pawlik et al. [39] randomized a group of low-weight newborn infants to receive an intravenous mixture of Clinoleic® plus Omegaven® or just a Clinoleic® parenteral emulsion. They found that, after 22 days of PN administration, 3 infants in the FO group developed cholestasis compared with 20 infants in the standard group (RR 0.18; 95 % CI, 0.055–0.56). The results of these publications agree with our results in the sense that they treat patients without PNALD, during a similar period of time and they have similar improvement in the LFTs. Our study goes beyond the previous ones by finding that increasing the dose of FO increases the improvement of ALT values independently of other factors.

Hepatic lipid accumulation is the result of an imbalance in *de novo* lipogenesis, FA oxidation, and/or TG secretion rates [40] and these mechanisms may also be involved in hepatic steatosis (with ALT and GGT elevation) occurring as a consequence of PN [17]. Increased intake of ω-3 PUFAs has been found to protect against fatty liver disease since it reduces hepatic lipogenesis and decreases the level of inflammation. Benefits have been noticed in patients with non-alcoholic fatty liver disease [41] and in animal studies on PNALD [21, 22, 40].

Among chronic liver diseases, the association between adipokines and non alcoholic fatty liver disease is well established. Adiponectine, the most abundant adipose-specific adipokine, decreases hepatic and systematic insulin resistance, and attenuates liver inflammation and fibrosis. That is why recent therapeutic strategies have focused on the indirect upregulation of adiponectin. In addition to its relation with chronic liver alteration, early data suggest that plasma levels of adiponectin are decreased in critical illness. Nevertheless, the detailed mechanisms underlying its hepato-protective functions remain largely uncharacterized [42–44].

No safety problems were detected in our patients since no significant differences were found when comparing serum levels of TG and coagulation parameters. The product labeling for the FO-based ILE indicates that it could increase bleeding risk, nevertheless authors such as Bays [45] and Harris [46] concluded that ω-3 PUFAs do not increase this risk. Results from Puder et al. [47] are in line with ours suggesting that FO has a limited effect on coagulation since the infants receiving FO had lower INR values and increased platelet counts compared with those treated with SO, moreover they point out that this increment in platelet counts suggests a possible improvement in liver function. The same authors [44] reported that the rate of hypertriglyceridemia in children receiving FO was lower than in children receiving SO; and TG levels in the FO group were lower (P =0.0002) and declined faster than in the SO group. Differences in levels and trends were statistically significant (P < 0.0001).

Some limitations can be attributed to this present study, such as the retrospective collection of data, even when the high degree of computerization reached in our hospital has allowed us to find almost every single piece of data that we wanted to study. Another possible limitation is that we did not study the amount of α-tocopherol administered. This could be important, and would be an interesting further avenue to explore since it is presently unclear whether the beneficial effects are caused by FO, α-tocopherol or by the exact mixture of the lipid components. On the other hand, histopathological measurements could have been undertaken to prove subclinical damage but, since they are not standard clinical procedures, they were not performed due to both ethical and financial concerns.

## Conclusions

Although the use of FO lipid emulsion as monotherapy is still questioned, some groups have shown that children receiving PN with Omegaven® monotherapy improve their LFTs and do not develop any signs of essential fatty acid deficiency or growth retardation [22]. Studying the effect of FO in adults, we have seen a clear improvement in some liver parameters (ALT and GGT) in a group of adult patients treated with PN during hospitalization. In addition, this improvement is larger when the dose of FO is increased. Since FO has been tried as a single lipid administered as PN in children and taking our results into account, it would be of interest to use higher doses of FO in those adult patients who develop liver parameter alterations during hospitalization with PN treatment. Randomized trials are needed to confirm this proposition.

## Abbreviations

ALT: Alanine aminotransferase
AP: Alkaline phosphatase
BIL: Bilirubin
FAs: Fatty acids
FO: Fish oil
GGT: Gamma-glutamyl transferase
ICU: Intensive care unit
LFTs: Liver function tests
LOS: Length of stay
PN: Parenteral nutrition
INR: International normalized ratio
PNALD: Parenteral nutrition associated liver disease
PLC: Platelets count
PUFAs: Polyunsaturated fatty acids
TG: Triglycerides
